# A systematic literature review: Real-time 3D reconstruction method for telepresence system

**DOI:** 10.1371/journal.pone.0287155

**Published:** 2023-11-15

**Authors:** Fazliaty Edora Fadzli, Ajune Wanis Ismail, Shafina Abd Karim Ishigaki

**Affiliations:** 1 Department of Emergent Computing, Faculty of Computing, Universiti Teknologi Malaysia (UTM), Johor Bahru, Johore, Malaysia; 2 Mixed and Virtual Environment Research Lab (mivielab), ViCubeLab, Universiti Teknologi Malaysia (UTM), Johor Bahru, Johore, Malaysia; National University of Sciences and Technology NUST, PAKISTAN

## Abstract

Real-time three-dimensional (3D) reconstruction of real-world environments has many significant applications in various fields, including telepresence technology. When depth sensors, such as those from Microsoft’s Kinect series, are introduced simultaneously and become widely available, a new generation of telepresence systems can be developed by combining a real-time 3D reconstruction method with these new technologies. This combination enables users to engage with a remote person while remaining in their local area, as well as control remote devices while viewing their 3D virtual representation. There are numerous applications in which having a telepresence experience could be beneficial, including remote collaboration and entertainment, as well as education, advertising, and rehabilitation. The purpose of this systematic literature review is to analyze the recent advances in 3D reconstruction methods for telepresence systems and the significant related work in this field. Next, we determine the input data and the technological device employed to acquire the input data, which will be utilized in the 3D reconstruction process. The methods of 3D reconstruction implemented in the telepresence system as well as the evaluation of the system, have been extracted and assessed from the included studies. Through the analysis and summarization of many dimensions, we discussed the input data used for the 3D reconstruction method, the real-time 3D reconstruction methods implemented in the telepresence system, and how to evaluate the system. We conclude that real-time 3D reconstruction methods for telepresence systems have progressively improved over the years in conjunction with the advancement of machines and devices such as Red Green Blue-Depth (RGB-D) cameras and Graphics Processing Unit (GPU).

## Introduction

Due to the high expense of three-dimensional (3D) reconstruction technology during the last two decades, virtual environments have been mostly restricted to research institutes, the medical, and other fields. Technological advancements in consumer-grade depth sensors have pushed this technology closer to the consumer market in recent years and expanded the research in this field exponentially. Research in 3D reconstruction is a subset of the computer vision area, and it is also significantly and positively connected to computer graphics research in several ways [1]. The goal of 3D reconstruction is to create a digital replica of a target object or the environment that exists in the actual world. This can be seen as numerous applications of 3D reconstruction have been discovered in a variety of disciplines, including health care [2], archaeology [3, 4] and telepresence [5–8]. 3D reconstruction is one of the fundamental requirements for the most immersive telepresence [9]. Telepresence has the opportunity that could benefit applications such as remote collaboration, entertainment, advertising, teaching, hazard site research, and rehabilitation [7, 10, 11].

Communication over long distances is essential to our everyday lives and jobs nowadays. Family and friends are relocating away from home to live and work in another location. International business excursions are something that many companies send their staff on. Video conferencing is a common communication mode that lets us instantly see and hear our friends and colleagues from anywhere. Remote expert advice through video is well-established in academia, health care, and industry. Despite its appeal, video is a relatively restricted means of communication compared to face-to-face encounters since the interlocutors are perceived as flat and remote. Besides that, video conferencing limits the sharing of a restricted view area and the fixed perspective of the local user [12], leading to weak interactivity and relatively poor user experience [13]. Hence, an immersive application such as telepresence represents a new generation of interactive services that provide end users with a rich and immersive experience. Telepresence technology enables a local user to connect with a remote user, and it is necessary to consider how the local user may capture and transmit his surroundings to the remote user. Currently, video calls face several drawbacks, including sharing a restricted view area and the fixed perspective of the local user [12]. The ability for the remote user to see an overview of the local user through advanced display technology could make it more efficient to overcome these limitations, allowing the user to experience a more expansive viewing experience compared to a conventional phone or monitor [14–17].

**Fig 1:**
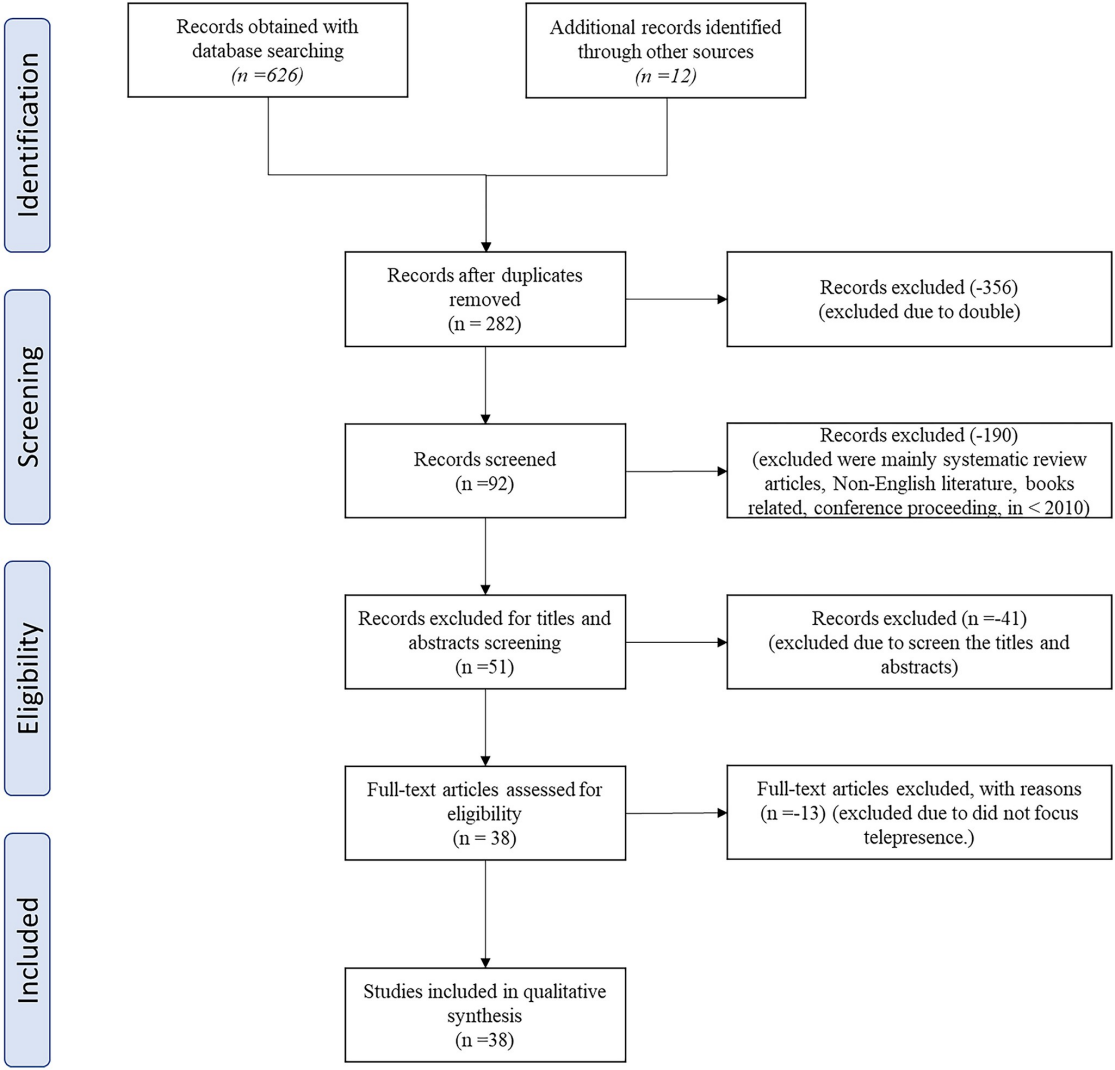
PRISMA flow of this report.

With the potential of integrating telepresence and 3D reconstruction technology, there is an opportunity to eliminate various constraints of traditional video-based communication mediums, and this advancement opens doors to new possibilities for remote collaboration [18, 19]. By utilizing realistic 3D user representations, modern telepresence systems enable individuals far apart to convene in virtual environments and interact with each other. However, it was challenging for researchers, programmers, or innovators to find a report presenting a survey on previous works, as few systematic reviews of 3D reconstruction for telepresence have been published in recent years. We ensure that it is essential to produce a comprehensive review to describe the most current methods and research findings in 3D reconstruction for telepresence systems. Therefore, this report examines, analyzes, and answers the research question. There are three primary advantages to the Preferred Reporting Items for Systematic Reviews and Meta-Analyses (PRISMA) Statement, which are as follows: First, identifies the research issue that leads to systematic research as shown in the PRISMA flow in Fig 1. Secondly, it helps to specify inclusion and exclusion criteria for systematic reviews. Thirdly, it attempts to analyze, within a specified term, a broad scientific literature database [20]. The PRISMA Statement can assist the authors in thoroughly searching for terms relevant to 3D reconstruction methods for telepresence systems. Through the analysis and summarization of many dimensions, we hope this report can provide researchers with a systematic and more in-depth understanding of real-time 3D reconstruction methods for telepresence systems and some references for this field of study. With metaverse’s ability to extend the physical world utilizing augmented reality (AR) and virtual reality (VR) technologies to allow users to seamlessly engage between real and simulated surroundings using reconstructed representation and holograms, we hope the technical breakthrough that has been covered throughout this report can be used as a guide to see the trend, strength, and weakness of implemented 3D reconstruction method.

## Background

Real-time 3D reconstruction can be defined as a process where the scene or the shape of an object in a physical world is captured, and the virtual representation of the scene or object is created in real-time. In computer vision, the term 3D reconstruction pertains to the process of restoring a 3D scene or target object within the scene from either a single view or multiple views of it.

**Fig 2:**
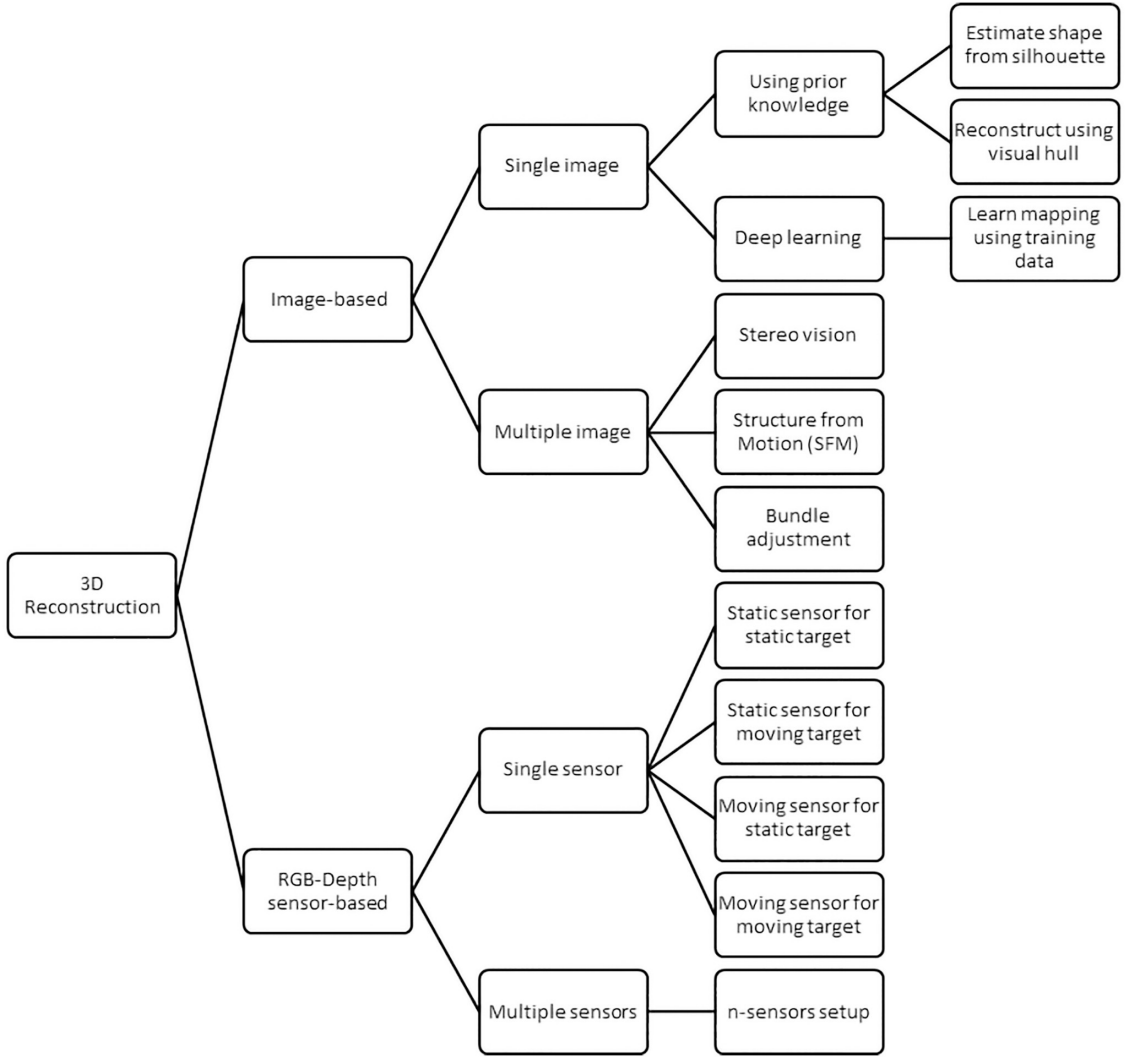
Classification of 3D reconstruction into image-based and RGB-Depth sensor-based.

A 3D representation of the entire scene, as classified in Fig 2, can be created using either a single photograph or multiple images captured from various perspectives as input. The past few years have witnessed multi-image 3D reconstruction with several traditional algorithms being presented, including stereo vision, SFM (structure from motion), and bundle adjustment. 3D reconstruction from a single image has been a long-standing and challenging task due to a large amount of information loss from two-dimensional (2D) images to 3D. With the advancement of neural networks and deep learning, it became clear that neural networks could be trained to learn the 3D structure of objects inside a single image [20]. Red Green Blue-Depth (RGB-D) sensors produce a detailed real-time measurement of 3D surfaces as a 4-channel signal. Colour channels in the RGB colour characterize the appearance of the surface, while a fourth depth channel offers local surface geometry metrics.

**Fig 3:**
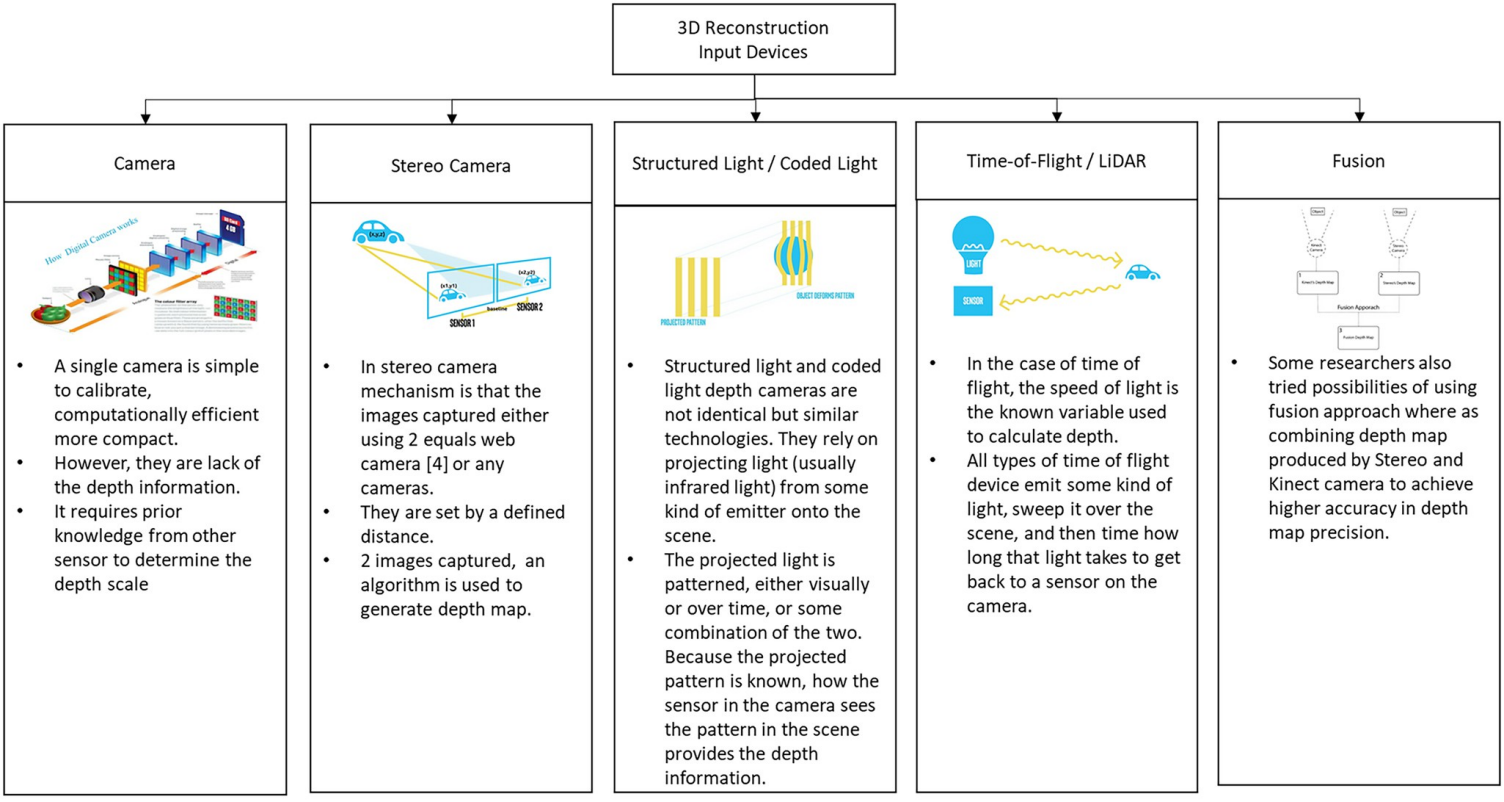
Type of sensor.

Since its initial introduction to the market ten years ago, RGB-D sensor hardware as can be seen in Fig 3, has played a crucial role in developing advanced mapping and 3D reconstruction systems. Its significance remains unchanged as it continues to contribute to these technologies. RGB-D cameras, such as the Microsoft Kinect, Intel RealSense, and Stereolabs Zed, are sensing systems that include an RGB camera, an infrared projector, and an infrared sensor. They may collect RGB data as well as the depth map at the same time [21]. 3D reconstruction with a single sensor can be accomplished in a variety of ways, including moving the sensor around a static target object or environment, capturing the target object or environment with a static or unmoving sensor, moving the target object in front of the static sensor, or moving the sensor around a moving object. While 3D reconstruction using multiple sensors required a suitable setup to set the position of the capturing depth sensors, considering the number of RGB-D sensors used and the field of view of the devices.

The fundamental technology that enables today’s structured light or time-of-flight-based depth cameras has existed for several decades. The release of these consumer-grade sensors that pack this technology into mass-produced devices with compact form has made RGB-D cameras a commodity available to a broader consumer. Several other devices, which include RGB-D cameras such as the Intel RealSense, PrimeSense Carmine, Google Tango, and Occipital’s Structure Sensor, have followed in the aftermath of the Kinect, which was introduced by Microsoft in 2010. These cameras are affordable, and their lightweight sensors can capture per-pixel colour and depth images at sufficient resolution and rapid frame rates. These characteristics put them ahead of even more expensive 3D scanning systems, which is especially important when creating solutions for consumer-grade applications. The potential of these new sensors in the field of visual computing has been swiftly recognized.

## Methods

Within this section, we explore the process involved in generating articles that are relevant to 3D reconstruction, specifically for telepresence systems. In this report, we employ the PRISMA technique, which comprises resources for systematic literature reviews (ACM Digital Library, IEEE Xplore, Springer Link, Scopus, and ProQuest journals). The inclusion and exclusion criteria, along with the review process steps (e.g., identification, screening, qualifying) and abstraction and analysis of information, are also carried out in compliance with the PRISMA approach.

### Defining the research questions

The primary objective of the systematic literature review (SLR) is to comprehend and recognize the 3D reconstruction method implemented in telepresence based on the research questions (RQs) and summarize it. The study topics and domains to match the performance of existing methods are also further employed. A total of three RQs were discussed as follows in order to achieve this objective:

- RQ1: What are the input data for the 3D reconstruction method?
- RQ2: What are the real-time 3D reconstruction methods implemented in telepresence systems?
- RQ3: How can the real-time 3D reconstruction method be evaluated for the telepresence system or application?

### Inclusion and exclusion criteria

A considerable measure of inclusion and exclusion criteria have been decided, as in Table 1. Regarding the literature type, we have selected article journals and conference proceedings that specifically concentrate on the study or design of 3D reconstruction methods or techniques employed in telepresence systems. Only available full-text literature was included. Review articles, book series, and chapters in a book have been excluded from consideration. Non-English publications were also withdrawn to avoid misunderstanding and confusion over the translated works. Finally, in terms of chronology (between 2010 and 2022), a period of thirteen years is chosen as an acceptable length of time long enough to grasp the evolution of research and related publications. Because the evaluation process concentrated on real-time 3D reconstruction for the telepresence system, articles published on 3D reconstruction that did not specifically target the telepresence system were removed from consideration.

**Table 1:** The inclusion and exclusion criteria.

Criteria	Inclusion	Exclusion
**Literature availability**	Full-text available	Full-text not available
**Type of paper**	Journal article and conference proceeding	Review articles, book series, and book chapter
**Language**	English	Non-English
**Timeline**	Between 2010 and 2022	Grey papers: These publications do not have bibliographical information such as date/type of publication, volume, and issue numbers or <2010
**Research question**	Papers answering at least two RQs	Papers that do not address the RQs

### Source and search study

The search was carried out using online scientific databases in the form of an online electronic search, relying on several journal databases. These online resources were selected because they were deemed to be the best relevant databases for delivering comprehensive information in the field of 3D reconstruction at the time of selection. Regarding peer-reviewed literature databases in electrical engineering, computer science, and electronics, IEEE Xplore gives web access to more than five million full-text documents from some of the world’s most highly cited journals. In academic literature, Scopus is one of the world’s largest and most well-regarded abstract and citation databases. The collection contains more than 40,000 titles from more than 10,000 foreign publishers, with almost 35,000 of these publications subjected to rigorous peer review. Scopus offers various forms, including books, journals, conference papers, and other materials. Springer also has many relevant records on 3D reconstruction for telepresence systems, which is an additional plus.

### Study selection

All studies were recorded in a Reference Manager System, and duplicates were eliminated when the search was completed. The remaining studies were then assessed using inclusion and exclusion criteria for the titles and abstracts. Where no judgment can be made on inclusion, the entire document has been read to give a final opinion.

### Data extractions

Data have been retrieved utilizing a data extraction form from the included studies. For this study, the form was specially constructed and included six data items, as seen in Table 2.

**Table 2:** Data extraction form items.

Data item	Descript
**Reference**	The title, author, year, type (Journal, Conference, Workshop)
**Aim**	The aim of the study, as the authors mentioned
**Input**	The input device or input data for the 3D reconstruction method
**Evaluation**	The description of how the 3D reconstruction for telepresence was evaluated
**Output**	The results of the 3D reconstruction method for telepresence
**Comments**	The author’s remark about the study

### Synthesis of results

Data analysis of the investigations was carried out after data extraction. The data gathered have been evaluated according to predetermined topics in the narrative format resulting from the research questions and discussed in the following topics:

1. Introduction of telepresence technology
2. 3D reconstruction methods for telepresence
3. Evaluation of 3D reconstruction method for telepresence.

### Comprehensive science mapping analysis

**Fig 4:**
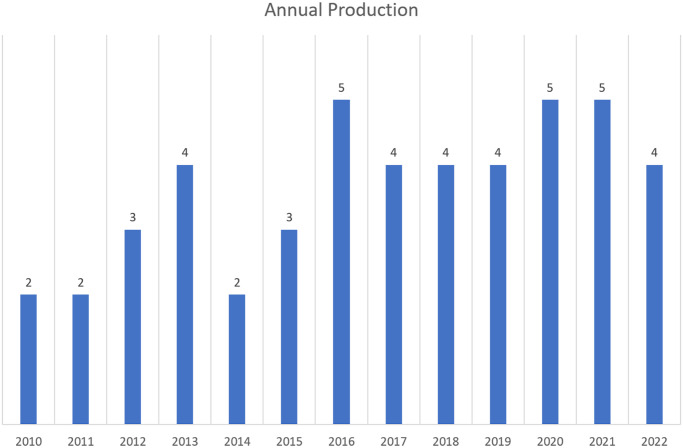
Annual production.

**Fig 5:**
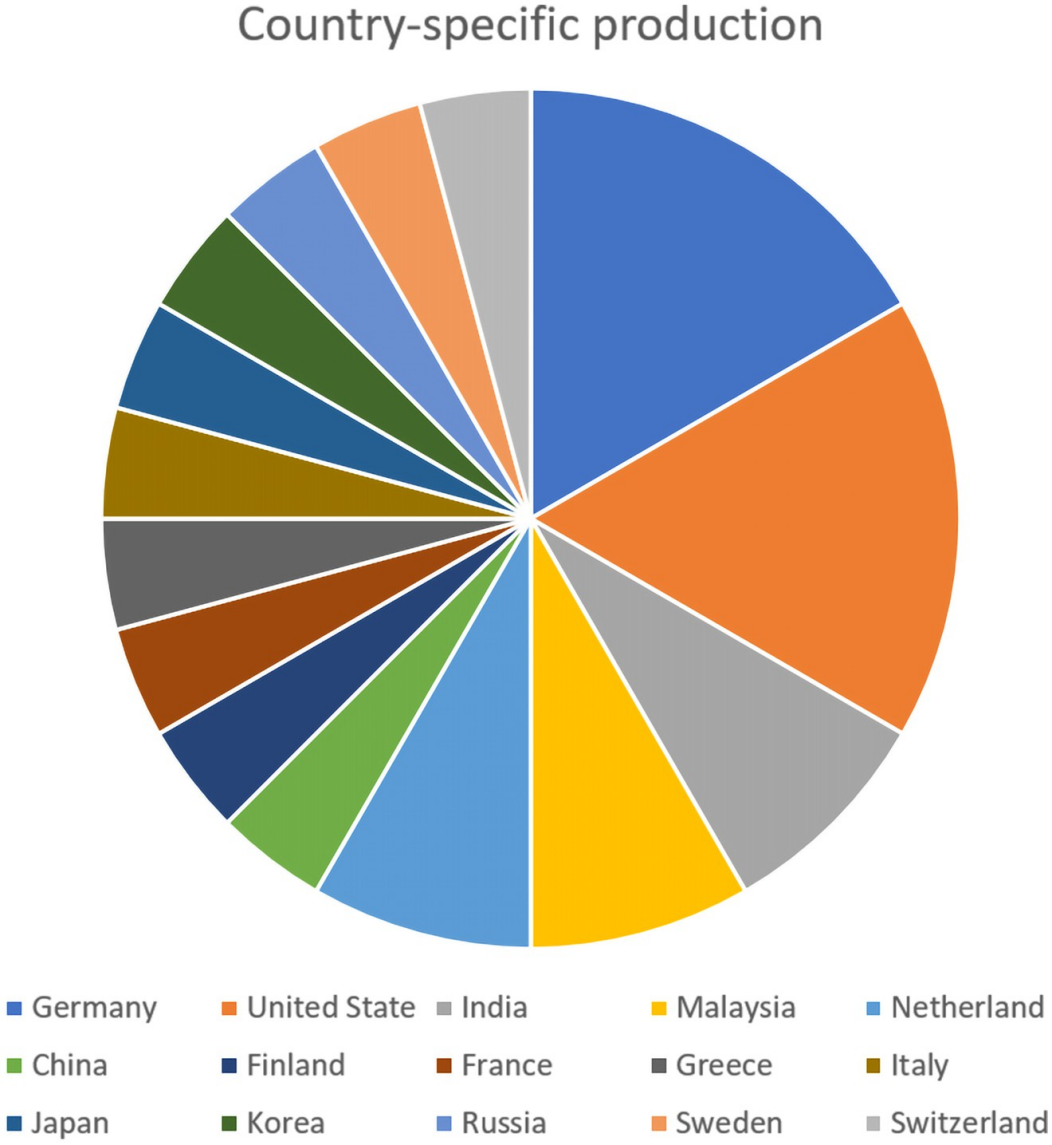
Country-specific production.

A comprehensive science mapping analysis, as referred to [22–24], was done to produce a bibliometric measurement of the included studies’ annual and country-specific production. The relationship between the production of research work concerning 3D reconstruction for telepresence systems and the year of publication is illustrated in Fig 4. As can be seen, the most significant number of these studies were published in 2016 and 2021, with five publications out of thirty-eight selected papers. The country-specific production of the included studies is shown in Fig 5. shows the geographical distribution of the included studies. Most of these studies (22, 48%) were published in North America (USA = 21, Canada = 1). After North America, the most common publication areas were Europe with 16 studies and 35% (UK = 4, France = 1, Netherland = 2, Germany = 4, Greece = 1, Finland = 1, Switzerland = 1, Sweden = 1, Italy = 1) and Asia with seven studies (India = 2, Malaysia = 2, Korea = 1, Japan = 1, Russia = 1, China = 1.

## Results

A total of 662 documents from the six top sites, ACM Digital Library, Google Scholar, IEEE Xplore, Springer Link, Scopus and ProQuest journals and candidate documents, have been collected. The number of overall publications on these platforms is not an indicator of their relevance but rather whether they capture the respective field. We analyzed each study to assess whether they suggest a 3D reconstruction method for a telepresence system to meet the mentioned limitations. Finally, for the survey, a total of 48 documents have been chosen. This systematic review has employed a standardized methodology (S1 Checklist).

### Telepresence technology

Marvin Minsky proposed the concept of "telepresence" to refer to the capability to control the tools of a remote robot as though using an individual’s real hands directly [25]. Remote manipulation paired with high-quality sensory feedback is the word used in this context to refer to remote manipulation. Later, Bill Buxton applied telepresence as a concept to the field of telecommunications [26]. In collaborative work, he distinguished between task space and person space and stated that "successful telepresence is reliant on the sharing of both in a high-quality manner." In the context of a shared environment with several users, Buxton et al. proposed that each teleconference participant be represented by a personal station equipped with audio and visual capabilities. Additional networked electronic whiteboards created a shared task environment [27]. Since then, significant progress has been made toward a shared person space with the concept of a shared and integrated environment for groups of people and tasks.

Visual communication systems of the modern era emphasize visual or spatial aspects and induce temporary disruption. This can influence the chances, the pace of a discussion and its meaning, how one perceives the other person and the interaction between them over time. The visual and spatial properties can be balanced by merging a 3D reconstruction and a display technology where both are free-viewpoint capable. Telepresence is a developing technology that attempts to deliver spatial immersion and virtual representation in a conventional non-physical environment. Several telepresence technologies have been recommended to provide end-to-end users with immersive and functional interactions [28]. The design of interactive environments using perspective views will enhance and integrate the co-space experience [29]. The use of audio-visual data and other stimuli to better understand co-location between users in the same virtual area is also being explored further.

The concept of telepresence combined with 3D reconstruction has motivated researchers for decades, albeit prototypes evolved slowly in the 1980s and 1990s due to technological constraints. Several cameras are deployed, and their imagery is constantly updated, including the moving user, to build a 3D reconstruction of the room-scale scene [30]. Telepresence began in 1994 as a system for collecting photometric and depth data from many fixed-stationary cameras. Virtualized reality [31] allows for the simulation of real-world events and their continuous movement to be captured in an image sequence; nevertheless, the movement of the real simulated image is not smooth and frequently disrupts.

On the other hand, Mulligan and Al [32] proposed a hybrid movement and stereo system to boost speed and power, even if the 3D environment must be obtained using remote location transmission. Towles et al. [33] stated that the sense of being there in the scene reconstruction is still feasible without relying on hardware or software technology. However, it was difficult to develop a complete Duplex device because of hardware and monitor configuration constraints. Tanikawa [34] proposed a technique in which photos of a person were collected from various cameras around the network and displayed on a revolving flat panel monitor in a range of image positions. However, due to the limitations of display technology, numerous positions overlap when a viewer is pushed around the viewing system. Kurillo et al. [35] acknowledged that an immersive VR system is designed for remote interaction and understanding of physical activity. To react to real-world or physical-world events, the configuration of a multi-camera system is required to execute 3D reconstruction.

As low-cost depth sensors such as the Microsoft Kinect became available, the number of studies and initiatives involving 3D reconstruction and its application in telepresence systems grew at a rapid pace. Beck et al. [6] introduce a novel immersive telepresence system that enables remote groups of users to interact in a shared virtual three-dimensional world created utilizing numerous Kinect devices. The purpose of telepresence is to create the illusion of being present or physically present with a remote individual. Telepresence can result from humans being projected in their natural size, 3D representations of the user or their environment, or mutual interaction between the remote site and the user. A variety of paradigms have been achieved, including a remote user who appears in a local location [36], a remote space appearing to expand beyond the local environment [4, 37], and a local user who is immersed in a remote area [38]. High-speed data transmission and real-time visualization of transmitted data are essential in a 3D telepresence system to transmit the 3D representation of the user or an environment and to ensure the interaction system is to provide immediate feedback [39]. Communication technology has developed rapidly with advancements in imaging and display technologies [40]. Various 3D communication systems are emerging, bringing more vivid and immersive experiences [4, 6, 41, 42].

#### Streaming or transmission of data

To accomplish the crucial success elements of an immersive telepresence experience, 3D telepresence systems have demanding standards for reconstruction, streaming speeds, and the visual quality of the obtained scene. For such a system to accomplish its intended purpose and be utilized effectively, multiple requirements must be met simultaneously. One of the most important criteria is low and dependable system latency; network latency is also essential for practical use. Similar requirements apply to videoconferencing services on 2D displays. If not met, audio/video synchronization could be impaired, unnatural breaks in visual continuity could occur, and the overall user experience could be reduced [43].

It is essential to minimize memory use in data transmission, as the greater the amount of data stored over an extended period, the larger the generated data set [44–46]. The potential solution is data compression [47–49]. Data compression is a technique that reduces the data size compared to its original size, making storage and transmission more efficient [50]. Compression data techniques are commonly employed in the telepresence system to ensure the data transmitted to the remote site arrives at the appropriate timestamp and in real-time [16, 51–53].

However, new source selection challenges have evolved for real-time telepresence with 3D model reconstruction across a network with constrained bandwidth. The first is shared bandwidth and real-time requirements. The bandwidth required to support a massive amount of video data from cameras is anticipated to surge, and the channel quality of each camera may affect the transmission rate [52, 53]. Nevertheless, the transmission latency is also critical to allow real-time interaction in VR systems, and as a result, the bandwidth demands and the real-time requirements need to be jointly assessed [54, 55].

For data transmission for the telepresence system, [56] uses a server that provides functionality to compress the voxel blocks and sends it to the client. The client listens for incoming volumetric data and assembles it once received. It has an exact copy of the server-side model for the telepresence system. [15] set the incoming data from the reconstruction client to first concurrently be integrated into the truncated signed distanced function (TSDF) voxel block model and then used to update the appropriate blocks and their seven neighbors in the Marching Cube voxel block representation in the negative direction. Maintaining such a collection for each connected client enables advanced streaming tactics required for a lag-free viewing experience and improves performance. [8] discovered that even after compression, depth images contribute to most network traffic, but colour images are comparatively small enough with jpeg compression and suggested adding temporal delta compression to the integrated lossless depth compression techniques increased compression ratios. All compression and streaming systems must balance bandwidth and computing speed.

#### Visualization

Another absolute requirement for a telepresence system is sufficient resolution. In this context, the resolution includes both spatial and angular resolution. Low spatial resolution can result in certain degrees of blur, which distorts the visual experience and makes it difficult or impossible to extract essential visual information, such as the individual’s facial expression [57]. Insufficient angular resolution may worsen, resulting in horizontal motion parallax disruption. In such a circumstance, visual phenomena include the crosstalk effect and sudden view jump, the mortal enemy of glasses-free 3D vision, as seen in [58]. However, high-end extremes should also be avoided, as the total system latency is also determined by a specific system’s processing demands and bandwidth utilization [43].

3D imaging and display technologies are significant technical elements for 3D communication. When constructing a 3D communication system, choosing appropriate 3D imaging and display technologies is essential. The 3D display methods can be categorized as binocular vision display [59], volume display [36], light field (LF) display [43, 60], and holographic display [61, 62]. Holographic display is a promising method for giving human eyes all the depth information [26–30]. Under coherent illuminations, computer-generated holography may reconstruct 3D intensity patterns from computer-generated holograms (CGHs) [63–65]. In recent studies [66–68], the holographic display for computer-generated objects has been developed. However, few studies on holographic displays process 3D data gathering into real-time display [40].

[69] mentioned 3D display technology that has been implemented in the telepresence system can be divided into two main devices, which are projectors [62, 70–73] and head-mounted devices (HMD) [45, 52, 58, 59, 74–77]. The projector device’s 3D display technologies are an on-stage hologram, autostereoscopic, and holographic projection, while HMDs can be classified into MR headsets and VR headsets. Before selecting the appropriate 3D display technology for a telepresence system, it is necessary to determine the number of users who will be displayed or projected and the number of users who will be perceiving the other user. The focus and purpose of the telepresence technology usage should also play a role in determining the optimal 3D display.

### Real-time 3D reconstruction methods for telepresence

Real-time 3D reconstruction is a crucial element for many immersive telepresence applications [9]; hence it is essential to identify which real-time 3D reconstruction methods are employed in telepresence systems. The general process involved in real-time 3D reconstruction can be identified as depth data acquisition, geometry extraction, surface generation, and fusion to generate a 3D model that represents point cloud or mesh data.

There are several methods of 3D reconstruction applied for telepresence systems that depend on the input data, such as for images or video data, which consist of the image frames, and there is an additional process required to obtain the depth data. However, traditional methods, such as the Shape-from-silhouette method, compute the surface of the visual hull of a scene object in the form of a polyhedron. As for 3D reconstruction using RGB-D sensors, the depth data obtained can be pre-processed or used directly as input data to compute the 3D representation of the target scene or object using a point cloud, mesh, or volumetric fusion approach. The list of included studies that have been analyzed to extract the information regarding 3D reconstruction methods for telepresence systems is as presented in Table 3.:

**Table 3:** Classified 3D reconstruction method for telepresence system of the selected primary studies.

Year	Study	Aim	Input	Evaluation	Method	Strength	Weakness
**2010**	Petit et al. [78]	To allow users to see themselves in virtual worlds by retrieving a 3D mesh of their environment in real-time, with textures attached.	Device: Multi-camera (8 webcams) Data: RGB images	Performance: refresh rates and latency	Visibility method: Shape-from-silhouette Method (EPVH)	The EPVH model is accurate visually. The level of detail is good enough to differentiate the user’s fingers. The application is robust to input noise. The resulting 3D model is watertight and manifold. Robust to network load change as the data transmitted could be resampled to avoid latency.	The approach only estimates normal velocity, not tangential velocity, on the mesh surface. Lack of data hinder mechanical interactions as user can modulate the force applied to a virtual object but cannot hold or grip anything.
**2010**	Moore et al. [79]	To investigate the deformation of 3D reconstruction models for various camera synchronization settings.	Device: Multi-camera (4 cameras) Data: Video frame	Performance: processing time for encoding of silhouette images. Visual quality: temporal qualities of acquisition	Visibility method: Shape-from-silhouette method (space carving process)	The 3D reconstruction and rendering processes benefit from separating texture and form, especially when a form is calculated in a centralized process closely aligned to the capture stages of the system.	In reconstruction with high delays, the effect on the model depends on which camera picture is delayed. Rotational direction impacts model deformation.
**2011**	Duckworth et al. [80]	To provide a better insight of the effect of synchronisation on the reconstruction of human body and its typical movements	Device: Multi-camera (6, 8, 16 cameras) Data: Video frame (pre-recorded datasets)	Visual quality: compare the quality of dataset results	Visibility method: Shape-from-silhouette Method (EPVH)	Synchronization errors that do not exceed a frame do not produce visible degradation.	Camera synchronization is key to accurately capturing human movement, so when frames are out of order, human shape reconstruction is impaired.
**2011**	Hauswiesner et al. [81]	Improvements in the robustness and performance of image-based visual hull rendering (IBVH) for virtual mirror and telepresence applications.	Device: Multi-camera (10 cameras) Data: RGB images	Performance: compares average kernel times of IBVH	Visibility method: Shape-from-silhouette Method (IBVH)	Frame- and view-coherence improvements resulting higher output resolution.	The motion detection algorithm, which triggers the redrawing of geometry fragments, is only a heuristic and may therefore introduce minor artifacts.
**2012**	Maimone and Fuchs [41]	A 3D acquisition system that can simultaneously capture and render a room-sized volume using a variety of depth cameras.	Device: Multi-RGBD sensor (4 Kinects) Data: RGBD images	Performance: measure the rendering rate. Visual quality: comparison of temporal noise	Volumetric method: TDSF for multi-camera	Reduced temporal noise over existing real-time systems that can capture room-sized scenes.	Unsynchronized sensors cause temporal inconsistencies at object edges during fast motion sequences.
**2012**	Maimone et al. [4]	Introduce a new telepresence system that collects and shows 3D scenes in stereo without the user wearing any tracking or viewing devices.	Device: Multi-RGBD sensor (4 Kinects) Data: RGBD images	Performance: measure the frame rate for display. Visual quality: comparison of temporal noise	Visibility method: Data merger algorithm	resolving issues related to interference, data merging and colour matching between units Improved calibration, data filtering, and data merger to increase image quality and implement in telepresence system.	some temporal noise artifacts are still present at the edges of objects where the depth camera alternates between providing a value and reporting no data at a given pixel.
**2012**	Zhang and Ho [71]	To report on a tele-immersive system of interaction constructed utilising 3D modelling techniques in real-time.	Device: Multi-camera (5, 24 cameras) Data: RGB images	Performance test: measure the speed of modelling.	Visibility method: Shape-from-silhouette	The octree depth influenced modelling time and follow-up performance indexes	Reconstruction is based on the intersection of all the cameras’ viewable ranges, limiting the effective acquisition space, thus the method cannot apply to long-distance physical movements.
**2013**	Alexiadis et al. [82]	A complete 3D realistic reconstruction system for moving people in real-time applications like 3D tele-immersion.	Device: Multi-RGBD sensors (5 Kinects) Data: RGBD images	Performance: measure the processing time and frame rate of enhancement method.	Volumetric method: Volumetric Signed Distance Function (SDF)	the volumetric reconstruction method which implemented in CUDA can run in real-time. can produce quite accurate and realistic results, even under the real-time constraints	Volumetric-based 3D reconstruction methods generate fewer triangles and vertices (depending on voxels) than the original 2D domain, thus full texture mapping was suggested.
**2013**	Zhao et al. [83]	Propose a method for multi-scale direction-aware filtering for 3D telepresence applications using Kinect.	Device: RGBD sensor (1 Kinects) Data: depth map	Performance test: computation time Visual quality: visual comparison results with four different data sets	Pre-process method: multi-scale filtering method	A multiscale direction-aware filtering approach that effectively addresses Kinect depth quantization. Reconstructed surfaces are closer to estimated ground truths and have better visual quality than common filtering method. Real-time filtering can be used in as pre-processing component in 3D telepresence applications.	Quantized strips have bumpiness if the filter window size is not proportional to the strip width.
**2013**	Beck et al. [6]	Introductory use of a depth correction volume to correctly calibrate individual depth cameras required for registering several depth sensors in a large environment.	Device: Multi-RGBD sensor (10 Kinects) Data: RGBD images	Performance: measure frame rates of application and network latency User testing: usability testing	Triangulation method: Warped triangle mesh method	demonstrated advances in registering multiple depth cameras based on the volumetric calibration of each involved Kinect sensor	the visual representation of remote users remains noisy as well as perforated due to occlusions
**2013**	Alexiadis et al. [57]	Real-time 3D reconstruction of moving objects for Tele-Immersion	Device: RGBD sensor (4 Kinects) Data: Point cloud	Performance: Reconstruction time, frame rate and number of mesh faces Visual quality: Comparative results with respect to the visual quality of the reconstructed model	Triangulation method: Mesh-based fusion	Noisy input and finite voxel resolution, inherent to volumetric methods, result in less artefacts and visually superior reconstruction outcomes. The proposed weighted smoothing method reduces reconstruction artefacts.	The triangular surface created by terrain SDCT is nonmanifold and has cracks and holes, hence the weighted smoothing method.
**2014**	Islam et al [84]	Present an unstructured lumigraph rendering method for high quality 3D depiction of a remote collaboration scenario (ULR).	Device: Multi-RGBD sensors (2 Kinects) Data: RGBD images	Performance test: Measure the real-time rendering speed	Triangulation method: mesh generation	Develop a dynamic proxy for unstructured lumigraph rendering to get a better, more detailed 3D proxy in real-time, leading to improved 3D scene rendering	Multiple Kinect projectors interference, causing holes on 3D scene at two Kinect intersections.
**2015**	Whelan et al. [85]	RGB-D Mapping is a 3D mapping technology that combines visual and shape-based alignment.	Device: RGBD sensor Data: Depth map	Performance: measure the sequential alignment comparison	Point-based method: RGB-D Mapping (sparse visual features and dense point clouds algorithm)	demonstrating the system’s capability to generate large-scale dense maps in real-time	the reliance on projective data association for camera pose estimation which limits the kinds of motion that our visual odometry frontend can handle
**2015**	Lu et al. [86]	To present tele-immersive system that allows people to communicate in a virtual world utilising body movements as well as words.	Device: RGBD sensor Data: Depth map	Performance test: Time performance for processing and rendering time.	Point-based method: 3D point cloud mapping	Integrated RGB-D systems, motion sensors, head-mounted displays, and networking into a real-time immersive telepresence system.	separate virtual view rendering component from the graphics tasks is due to its mixture of input from multiple resources.
**2015**	Roberts et al. [74]	An end-to-end solution that blends video conferencing with virtual worlds.	Device: Multi-camera (8 cameras) Data: video	Performance test: Time performance for processing frame rate.	Visibility method: Shape-from-silhouette Method (EPVH)	Two distribution models were proposed, each fitting a different approach to video-based reconstruction	Background segmentation against a moving background has only been tested using pre-recorded video. Required bandwidth and thus the latency are dependent on number of cameras, which is dependent on placement
**2015**	Almeida et al. [87]	An online incremental 3D reconstruction system for telepresence and HMI applications.	Device: RGBD sensor (1 Kinects) Data: RGBD data	Visual quality: the visual quality of the reconstructed model	Triangulation method (Crust algorithm)	introduced free viewpoint system framework to generate view dependent synthesis based on scene 3D mesh model. New incremental version of Crust technique adds new vertices to an existing surface without recomputing previous generated meshes. Topological incremental reconstruction approach based on confidence measures prevents redundant data information computation.	The body to be reconstructed should be segmented from background static areas using a motion filter
**2016**	Escolano et al. [42]	3D reconstructions of a full room, including people, furniture, and items, in real-time utilising modern depth cameras.	Device: RGBD sensor (8 custom RGBD sensor) Data: RGB and NIR image	Performance: computational time latency Visual quality: the visual quality of the reconstructed model User test: User study	Volumetric method	Dual-GPU implementation where frame-to-frame motion estimation does not require the feedback from the second GPU, so two GPUs can run in parallel. Combine a new capturing technology with mixed reality displays to offer 3D remote collaboration.	The system requires high amount of high-end hardware. colour artifacts during texturing process caused by extreme occlusions in the scene. reconstruction of smaller geometry such as fingers generated artefacts such as missing or combined surface.
**2016**	Fairchild et al. [75]	A telepresence technology and an application to facilitate collaborative space exploration	Device: Multi-camera (2 cameras) Data: video	Performance: computational time Latency Visual quality: the visual quality of the reconstructed model	Visibility method: Shape-from-silhouette Method (EPVH)	This article introduces a new background segmentation method that lets a person be captured without their surroundings. A new texturing method removes visible lines at polygon intersections without blending.	The viewpoint-based blending technique only effective when the viewer looks at the reconstructed mesh from near a camera view.
**2016**	Pejsa et al. [70]	Involves projected AR to provide life-size co-presence between two remote individuals.	Device: RGBD sensor (2 Kinects) Data: RGBD data	Performance: computational time latency Visual quality: visual quality feedback User test: User study	Triangulation method: Mesh generation	The capture process will acquire people moving around the room as well as dynamic objects during calibration.	Projections of virtual copies suffer from visual artifacts due to low depth sensor resolution and occlusion issues.
**2016**	Zioulis et al. [51]	3D reconstruction of users in a pre-authored 3D environment.	Device: RGBD sensor (4 Kinects) Data: RGBD images	Performance: computational time latency	Volumetric method: Fourier Transform-based	The methods were implemented using CUDA, to exploit the parallel processing capabilities of the GPU and perform near real-time.	the signalled acquisition method, synchronization issues were observed only during very fast user’s motions
**2016**	Dou et al. [88]	A novel multi-view performance capture and reconstruction pipeline	Device: multiple RGBD sensor (24 trinocular) Data: RGB and Depthmap	Performance: computational time frame rate Visual quality qualitative comparisons among different state of the art methods	Volumetric method	captured a variety of diverse and challenging nonrigid moving scenes. reconstruction algorithm can deal with extremely fast motion	The frame-to-frame correspondences would be incorrectly approximated or the nonrigid alignment would fail to converge if the frame rate is too low or frame-to-frame motion is too large. Missing depth data can cause large segmentation errors that affect visual hull estimate. any small nonrigid alignment errors can cause slight over smoothing of the model at times
**2017**	Joachimczak et al. [52]	MR device that wirelessly feeds 3D reconstructions of people and objects to Microsoft’s HoloLens head mounted display at steady frame rates.	Device: RGBD sensor (2 Kinects) Data: RGBD data	Performance: computational time	Point -based method: Point Cloud Library (fast mesh algorithm)	achieve high speeds and low polygon counts by using Point Cloud Library organized fast mesh algorithm. transmit high-resolution texture data and low-polygon 3D models to HoloLens to achieve high reconstruction frame rates.	lower quality 3D models due to low polygon counts and subsequent 3D reconstruction data lost or stalled because of unreliable wi-fi connection
**2017**	Tan et al. [19]	Use a pair of consumer-grade RGBD cameras to develop telepresence systems using robust and real-time head reconstruction technology.	Device: 2 RGBD sensor Data: RGBD images	Performance: latency Visual quality: the visual quality of the reconstructed model	Point-based method: Iterative refinement method	have an automatic registration process, that requires minimal input from the user. maintain a temporal boundary to minimize face silhouette visual artefacts caused by missing data and imprecise depth acquisition.	the usage of commodity sensors limits the display resolution and RGB quality captured. The two RGBD sensors may not register data if the user’s face cannot be identified in poor lighting. the boundary tracking method cannot handle scenes with excessively complex background
**2017**	Komiyama et al. [15]	Intuitive telepresence system using a head-mounted first-person camera and multiple ambient depth sensors.	Device: RGBD sensor (4 Kinects) Data: RGBD data	User test: Pilot study	Point-based method	depth data from four Kinect sensors is combined into a point-cloud space with three-dimensional positions.	Since each point has a 3D position, transmitting in point cloud format increases data size.
**2017**	Su et al. [53]	A novel system that enhances distant user interaction with dynamic virtual 3D environments	Device: RGBD sensor (5 Kinects) Data: Images	Performance: measure alignment error Visual quality: evaluate quality of the rendering result	Point-based method (sphere fitting algorithm)	Calibration methods can accurately and efficiently combine multi-views into a unified coordinate system	The backside of the remote user in the reconstructed scene is missing due to only using one single camera at the remote places
**2018**	Parikh and Khara [76]	An environment in which the user can engage with the other party via a wearable mixed reality headset	Device: RGBD sensor (8 trinocular cameras) Data: RGBD images	Performance: latency	Visibility method: Shape-from-silhouette method	Temporal consistency can separate and construct a more consistent object model.	Many users can be accommodated per workspace by either increasing the bandwidth, processing power and improving the compression algorithm
**2018**	Ruchay et al. [89]	Analyze depth data from many Kinect sensors to estimate 3D object reconstruction accuracy.	Device: RGBD sensor (4 Kinects) Data: RGBD images	Performance: root mean square error (RMSE) of measurements	Point-based method: Point cloud fusion	Median filtering recovers incomplete regions. calibration with derived distortion parameters is more accurate than Kinect’s built-in mapping.	The accuracy of device used is not very good and degrades with distance
**2018**	Bell et al. [90]	A novel pipeline for 3D video recording, encoding, compression, decompression, display, and interactivity.	Device: RGBD sensor (structured light scanner – projector & camera) Data: RGBD data	Performance: frame rate	Triangulation method	Digital fringe projection reconstructs 3D shapes using phase-shifted fringe patterns instead of intensity information which has more resolution than standard structured light methods	The 3D reconstructions from lossy encoded image incur some reduction in measurement accuracy
**2018**	Du et al. [16]	Combining several video textures onto dynamic meshes in real-time with virtually imperceptible view transitions	Device: Multiple RGBD sensor Data: multi-view video stream	Performance: frame rate number of vertices computational time Visual quality qualitative comparisons among different state of the art methods	Triangulation method	Fusing several video textures onto dynamic meshes with nearly undetectable view transitions in real-time	The system suffers artifacts resulting from the extruded triangles reconstructed during very fast motion. insufficient reliable colours cause missing texture field
**2019**	Stotko et al. [91]	With virtually undetectable view transitions, interactively blend video textures onto dynamic objects.	Device: RGBD sensor (Kinect or smartphone) Data: RGBD images	Performance: computational time bandwidth latency Visual quality visual quality of the reconstructed 3D models User test: usability testing	Volumetric method: TSDF	the first thread-safe GPU hash map data structure that assures concurrent insertion, retrieval, and removal while maintaining key uniqueness required by existing voxel block hashing techniques.	move and turn rapidly might cause high angular and linear velocities and motion blur which then cause reconstruction more susceptible to misalignments.
**2019**	Bell and Zhang [17]	Novel platform for real-time wireless 3D video communications between mobile devices	Device: RGBD sensor (smartphone) Data: mobile 3D video	Performance: frame rate bandwidth latency	Triangulation method: 3D range geometry encoding method	When reconstruction quality can be reduced, stronger H.264 compression can reduce video bit rate.	data resolution and frame rate, connection speeds of 1-2 Gbps are commonly needed to send 3D geometry video in real-time. These speeds are exceedingly demanding and can sometimes only be achieved over a wired network connection.
**2019**	Córdova-Esparza et al. [92]	Low-cost visual telepresence systems with depth sensors	Device: RGBD sensor (4 Kinects) Data: RGBD images	Performance: latency True Positives (TP) rate Visual quality visual quality of the reconstructed 3D models	Point-based method: point cloud extraction and fusion	The many RGBD cameras used allow for an omnidirectional view, contrary to other telepresence methods.	the system is currently limited to 3D point cloud visual-only information
**2019**	Teo et al. [58]	A new system with two local and one remote user using AR HMDs. And tools to design a room with a remote user.	Device: Camera (360 camera) Data: 360° video	Performance: refresh rate User test: user study (collaborative task completion time) user experience	Point-based method: Photogrammetric 3D spatial data	system allows switching between 360 and 3D modes to introduce variation to solve collaborative tasks	Hardware limitations and a task that largely relied on colour and shape could have increased noise. the visual cue offset and 3D scene quality that was directly influencing the user experience
**2020**	Cho et al. [93]	Comparison of actor volumetric capture with 2D footage and 3D avatar created by pre-scanning the actor	Device: RGBD sensor (1 Kinects) Data: RGBD images	Performance: computational time latency User test: user study (presence)	Triangulation method: polygonal mesh	the highest sense of social presence was achieved with volume-capture when performing dynamic tasks	Volume-capture issues like blurry textures and unstable boundaries are partly due to Kinect SDK’s limitations and the low-resolution voxel grid. audio-video discrepancy caused by volume-capture latency
**2020**	Wang et al. [94]	A new telepresence compression strategy based on 3D point clouds is proposed.	Device: RGBD sensor (1 Kinects) Data: RGBD images	Performance: Time performance for processing Visual quality: the visual quality of the reconstructed model	Point-based method	The robust colour-based Bayesian framework reduces spatial redundancy and motion vector estimation reduces temporal redundancy between frames.	the number of 3D points depends on the size of the actual frames
**2020**	Young et al. [55]	Present Mobileportation, a mobile telepresence prototype that allows seamless transitions between freely shared 3D environments.	Device: Hybrid sensor Data: RGBD data	Performance: frame rate Time performance for processing User test: user evaluation	Point-based method	Real-time frame rates were obtained for reasonably sized environments to give users the smooth, seamless experience they’re used to from conventional videoconferencing solutions.	Due to the real-time requirement, complicated storage solutions like an octree were not possible, hence numerous duplicate points were captured, stored, and rendered.
**2020**	Laskos al. [77]	A real-time 3D reconstruction solution for the human upper torso in mixed reality applications employing only one depth camera on the capture side.	Device: RGBD sensor (1 Zed Mini) Data: RGBD images	Performance: frame rate Execution time	Triangulation method: mesh generation	Marching Cubes algorithm was used after quantizing the point cloud	The data size depends on the point cloud vertices after user extraction, the mesh generation algorithm step, the texture image resolution and format, and the frames per second rate.
**2020**	Du et al. [95]	Introduce DepthLab, a software library for depth-based UI/UX paradigms.	Device: RGBD sensor (smartphone) Data: Depth map	Performance: completion time	Triangulation method: mesh generation	introduce interaction modules and real-time algorithms building upon three data structure representations of depth	DepthLab enables geometry-aware AR experiences on phones with and without time-of-flight sensors, thus dynamic depth design yet to be explored.
**2020**	Fadzli et al. [59]	To explore real-time 3D reconstruction for MR telepresence.	Device: RGBD sensor (1 Kinects) Data: RGBD image	User test: usability testing	Point-based method	robust 3D reconstruction method integrated into collaborative MR interface that enable local and remote user to work on collaborative task	MR wearable headset was felt heavy on the user when performing interaction. Users had to cross their eyes to see MR view properly due to hardware distortion.
**2021**	Yu et al. [96]	A 3D Telepresence system allows users to interact with each other and compares both point cloud reconstruction or virtual character to identify the differences between user representations and their fit in the reconstructed environments	Device: RGBD sensor (4 Azure Kinects) Data: Depth images	Performance: latency	Point-based method: point cloud extraction	All image processing and network components are distributed, multi-threaded C++ and CUDA data-flow systems to meet performance and latency requirements. native Unity3D data-flow engine was integrated for low-latency, high-throughput streaming	did not use sophisticated pose fusion algorithms to combine avatar poses from multiple cameras
**2021**	Cha et al. [97]	presented a real-time egocentric 3D capture system as a step toward a fully mobile telepresence system which makes use of visual and inertial sensors that are either easy to embed into or are already present in commonly worn personal accessories such as eyeglasses, watches, and shoes.	Device: eyeglasses-mounted cameras and a few visual-inertial sensors	Performance: accuracy Visual quality qualitative comparisons of the reconstructed 3D models	Template-based reconstruction	use the SMPL parametric body model to represent the body shape and pose	The system only tracks the user’s limbs and cannot detect topological or textural changes in the body model or model interactions with the environment. If a 3D joint is detected erroneously, the temporal orientation network propagates the error.
**2021**	Yu et al. [56]	introduce Magnoramas that allow the flexible extraction, transformation, and annotation of a region of interest (right) inside the real-time captured point cloud as well as can be interactively positioned, rotated, and scaled by the user.	Device: RGBD sensor (3 Azure Kinects) Data: Depth and infrared images	User test: user study	Point-based method: point cloud extraction	a novel interaction method for selecting and extracting a region of interest of reconstructed point cloud that the user can subsequently scale and transform inside the virtual space	the quality of the real-time captured point cloud introduced artifacts. the user’s HMD is not visible in the point cloud due to the high reflection coefficient of the transparent display
**2021**	Rasmuson et al. [98]	demonstrate a semiautomatic real-time pipeline for capturing and rendering free-viewpoint video utilizing passive stereo matching on a desktop computer with inexpensive web cameras.	Device: Webcams (6 Logitech C922 webcam) Kinects) Data: video streams	Performance: Reconstruction time frame rate Visual quality: the visual quality of the reconstructed model	Visibility method: Shape-from-silhouette	achieve usable free-viewpoint video in under 15ms with a fraction of the resources and simple setup allows nearly anyone with a few webcams to make material appropriate for AR and VR head-mounted displays and holographic screens in real-time.	The resolution of the reconstructed geometry has diminishing returns on the perceived quality of the final view. Without global surface reconstruction, triangles with oblique angles that protrude in many directions and poor reconstruction must relied on filter.
**2021**	Bortolon et al. [99]	propose capturing nearly synchronised frame streams from multiple moving handheld mobiles for dynamic object 3D reconstruction.	Mobile phone’s depth camera (6 mobile phones) Data: Depth and infrared images	Performance: measure the throughput and latency. Average and standard deviation time difference between consecutive frames received.	Volumetric method: Marching Cube	The system can capture almost synchronous frames to reconstruct a moving person’s 3D skeleton and mesh.	mobile poses are sometimes inaccurate, and that data may undergo variable delays
**2022**	Song et al. [100]	present the concept of Dynamic 3D View Sharing, which complements the views of a 3D reconstruction system by the dynamic view of the user’s HMD.	Device: RGBD sensor (4 Azure Kinects) Data: Depth and infrared images	Performance test: accuracy	Point-based method: point cloud extraction	present a markerless calibration method. as the dynamic 3D sensor is free from the limitations of the static cameras, the lost information can be picked up by viewing at a closer distance or alternative angle	although the 3D capturing system was able to reconstruct most of the scene, there are still many gaps within the fused point cloud
**2022**	Fadzli et al. [61]	introduces a real-time 3D reconstruction of a human captured using a depth sensor and has integrated it with a holographic telepresence application	Device: RGBD sensor (2 Kinects) Data: Depth and infrared images	Performance test: processing time processing rate average number of vertices and triangles.	Volumetric method: Marching Cube	the approach is compact and readily manages data, which can benefit telepresence, which requires instant transmission and fast and compact data structures.	The application runs in real-time until surface generation, excluding texture mapping.
**2022**	Montagud et al. [101]	Towards socialVR: evaluating a novel technology for watching videos together.	Device: RGBD sensor (4 Kinects) Data: RGBD images	User test: User study	Triangulation method: Mesh-based fusion	To achieve coherent volumetric capturing, each sensor’s RGB-D frames are merged in synchronize. After capturing, a background removal technique isolates geometry from colour information required for the user’s 3D representation.	limitations in terms of technological aspects, such as the latency, fluidity and resolution of the end-user’s reconstruction
**2022**	Fischer et al. [8]		Device: RGBD sensor (Azure Kinects) Data: RGBD images	Performance: measured the time needed for filtering, compression, and rendering the frame rate. User test: user study		Modular low-latency multi-camera RGB-D streaming pipeline, including filtering, denoising, and compression. Custom splat- and mesh-based point cloud rendering solutions	lack of a dedicated point cloud/mesh fusion process Even when registered correctly, rendering multiple point clouds or meshes of the same object individually causes seams or artefacts.

#### Visibility method: Shape-from silhouette

**Fig 6:**
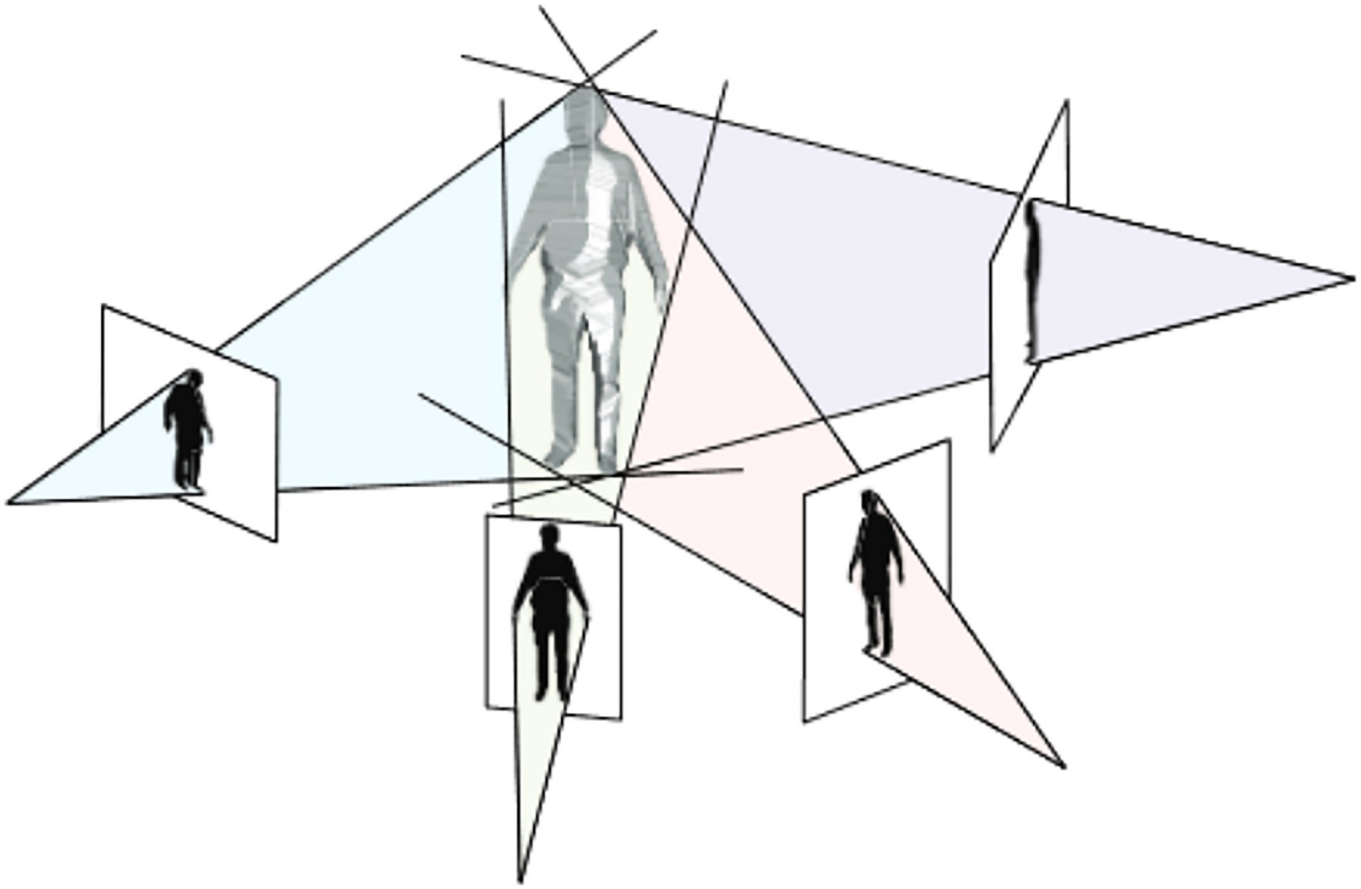
Visual hull from 4 views [11].

The shape-from-silhouette approach creates a shape model called the visual hull to obtain a three-dimensional geometric representation of objects and people in the acquisition space. This method generates shape models for use in subsequent stages of the process, such as texture mapping or interaction in real-time. The visual hull is defined geometrically as the intersection of the viewing cones, which are generalized cones whose apices are the projective centers of the cameras and whose cross-sections overlap with the silhouettes of the scene, as illustrated in Fig 6. When piecewise-linear photo contours for silhouettes are considered, the visual hull is transformed into a regular polyhedron. Although a visual hull cannot model concavities, it can be efficiently computed, resulting in a very excellent approximation of the human shape. The disadvantage of shape-from-silhouette techniques, according to [102], has mentioned not being capable of reconstructing concave regions adequately.

The EPVH algorithm has the unique ability to retrieve an exact 3D geometry that refers to the silhouettes obtained. This is a significant advantage over other algorithms. When models need to be textured, this is an important feature because it allows Textures from silhouettes to be mapped directly on a 3D model, which is very useful. The 2D polygonal outline of the object of the scene is obtained for each view. A discrete polygonal description of silhouettes of this type results in a unique polyhedron representation of the visual hull, the structure of which is retrieved using the EPVH algorithm. In order to execute this, three measures need to be taken. For starters, a specific polyhedron edge subset is generated, the viewing edges, which are the visual edges induced by viewing lines of contour vertices. A second step involves recovering all the other edges of the polyhedron mesh via a sequence of recursive geometric deductions. The positions of vertices that have not yet been computed are gradually inferred from those that have already been computed, with the viewing edges serving as a starting set of vertices. The mesh is traversed repeatedly in a consistent manner in the third step to identify each face of the polyhedron.

#### Volumetric method: Truncated signed distanced function (TSDF)

The volumetric surface representation format based on the TSDF displays an environment in 3D using a voxel grid where every voxel records the nearest area’s distance. This method has been used in current depth camera-based environment mapping, and location systems use the representation widely.

**Fig 7:**
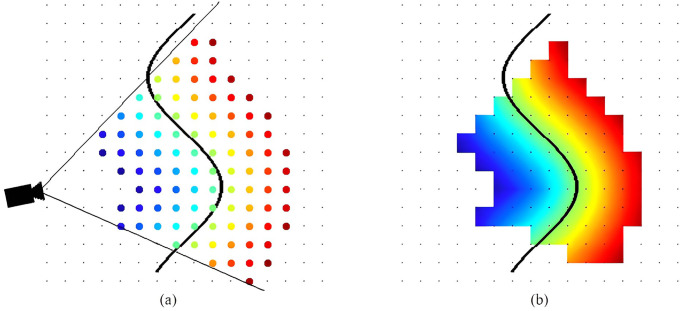
Truncated signed distance function (TSDF). (a) and the TSDF that was interpolated (b). Every voxel is represented as a dot in the grid, while the black line represents the surface. Positive distances are indicated by the colour blue to green; negative distances are indicated by the colour green to red. [103].

An n-dimensional world is represented by an n-dimensional grid of voxels of equal size. A voxel’s location is specified by its center. There are two significant values for each voxel. To begin, *sdf*_*i*_(*x*) is the signed distance between the voxel center and the closest object surface in direction of the current measurement. Values are defined to be positive in front of an object in free space. Distances behind the surface, which is within the object, are negative. Likewise, each voxel has a weight, *w*_*i*_(*x*) that is used to quantify the uncertainty associated with the corresponding, *sdf*_*i*_(*x*). The subscript *i* indicates that this is the *i* ’th observation. *sdf*_*i*_(*x*) is defined as in Fig 7 and the following equation.

$${sdf}_{i}\left(x\right)={depth}_{i}(pic(x))-{cam}_{z}(x)$$

*pic*(*x*) is the depth image projection of the voxel center *x*. Thus, *depth*_*i*_(*pic*(*x*)) denotes the depth measured between the camera and the closest object surface point *p* on the ray crossing *x*. Consequently, *cam*_*z*_(*x*) is the distance along the optical axis between the voxel and the camera. As a result, *sdf*_*i*_(*x*) is also a distance along the optical axis.

Truncated (±*t*) SDF is advantageous since vast distances are irrelevant for surface reconstruction, and a value range constraint can be used to reduce memory usage. *tsdf*_*i*_(*x*) is the abbreviation for the truncated variant of *sdf*_*i*_(*x*).


$$t{sdf}_{i}\left(x\right)=\frac{W_{i-1}\left(x\right){TSDF}_{i-1}(x)+w_{i}(x){tdsf}_{i}(x)}{W_{i-1}\left(x\right)+w_{i}\left(x\right)}$$

$$W_{i}\left(x\right)=W_{i-1}\left(x\right)+\;w_{i}\left(x\right)$$

**Fig 8:**
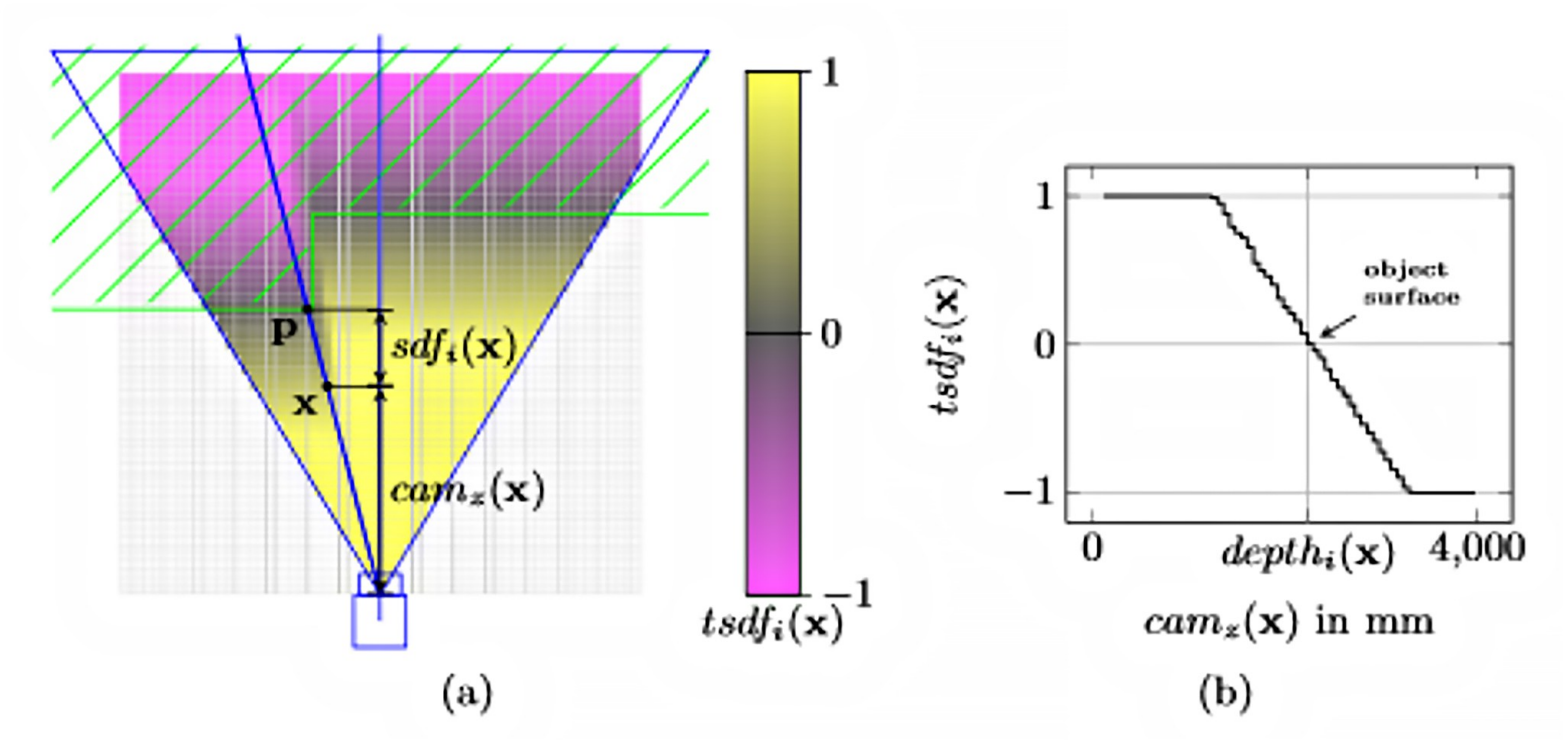
Reference image of TSDF algorithm. a) The camera’s field of vision, optical axis, and ray (blue), as well as the TSDF grid (unseen voxels are white). (b) A TSDF sample was taken along the ray [104].

Fig 8(a) shows that the *tsdf*_*i*_(*x*) of the voxel grid are expressed using colour. The TSDF is sampled along a viewing ray in Fig 8(b). Observations from multiple views can be integrated into a single TSDF to integrate data from multiple perspectives to increase accuracy or to fill in missing spots on the surface. This is accomplished through weighted summation, which is often accomplished using TSDF iterative updates. *tsdf*_*i*_(*x*) signifies the integration of all observations, *tsdf*_*i*_(*x*) with 1 ≤ *j* ≤ *i*. *W*_*i*_(*x*) quantifies the uncertainty of *TSDF*_*i*_(*x*)). The following update phase incorporates a new observation for each voxel x in the grid. The grid is initialized with *TSDF*_*i*_(*x*) = 0 and *W*_0_(*x*) = 0.

[105] framework’s server executes scene reconstruction and employs the internal data structure Voxel Block Hashing (VBH) [106] for scene representation. VBH will only save a voxel block if at least one of its voxels is within the TSDF’s truncation band. A spatial hash function addresses the blocks, which converts a 3D voxel position in world space to a hash table entry. [107] implements VBH to hold the fused colour and 3D depth information for volumetric representation with hashes.

#### Triangulation method: Mesh generation

To some degree, the purpose of 3D reconstruction is to make the reconstructed scene or object visible. Through 3D reconstruction methods, a group of 3D dot sets can be generated; however, this method cannot reflect surface details. As a result, these spatial coordinates should be triangulated, and a simulated surface composed of multiple triangles can be employed to approximate the actual surface.

It is possible to achieve this using a collection of 3D point sets and applying the 3D reconstruction algorithms. However, the surface details cannot be replicated through this method. As a result, it necessitates the triangulation of these spatial coordinates, and the usage of a simulated surface comprised of multiple triangles to resemble the actual surface can be accomplished. This process establishes a networked structure for the scattered 3D point sets. The object’s 3D model will be created using triangular plane clips following triangulation. We can now retrieve the actual 3D model by extracting the texture from the image and projecting it onto the 3D model.

The direct meshing of point clouds is possible using Delaunay triangulation and its variants [21]. These methods are susceptible to noise and inconsistencies in the distances between points. Maimone and Fuchs [4, 41] independently construct triangle meshes for multiple cameras by connecting adjacent range image pixels. The meshes are not blended together but are rendered separately. The frames are then combined. Alexiadis et al. [57] take the concept further by merging triangle meshes before rendering. While these techniques can achieve high frame rates, the output quality could be improved.

Delaunay’s Triangulation is one of the most frequently used triangulation methods since it is characterized by optimality. Delaunay presented it for the first time in 1934. There are three primary ways for Delaunay’s Triangulation: the incremental method (incremental insertion), divide algorithm (segmentation-merger algorithm), and triangulation growth algorithm abandoned in the mid-1980s. The other two techniques are particularly common.

**Fig 9:**
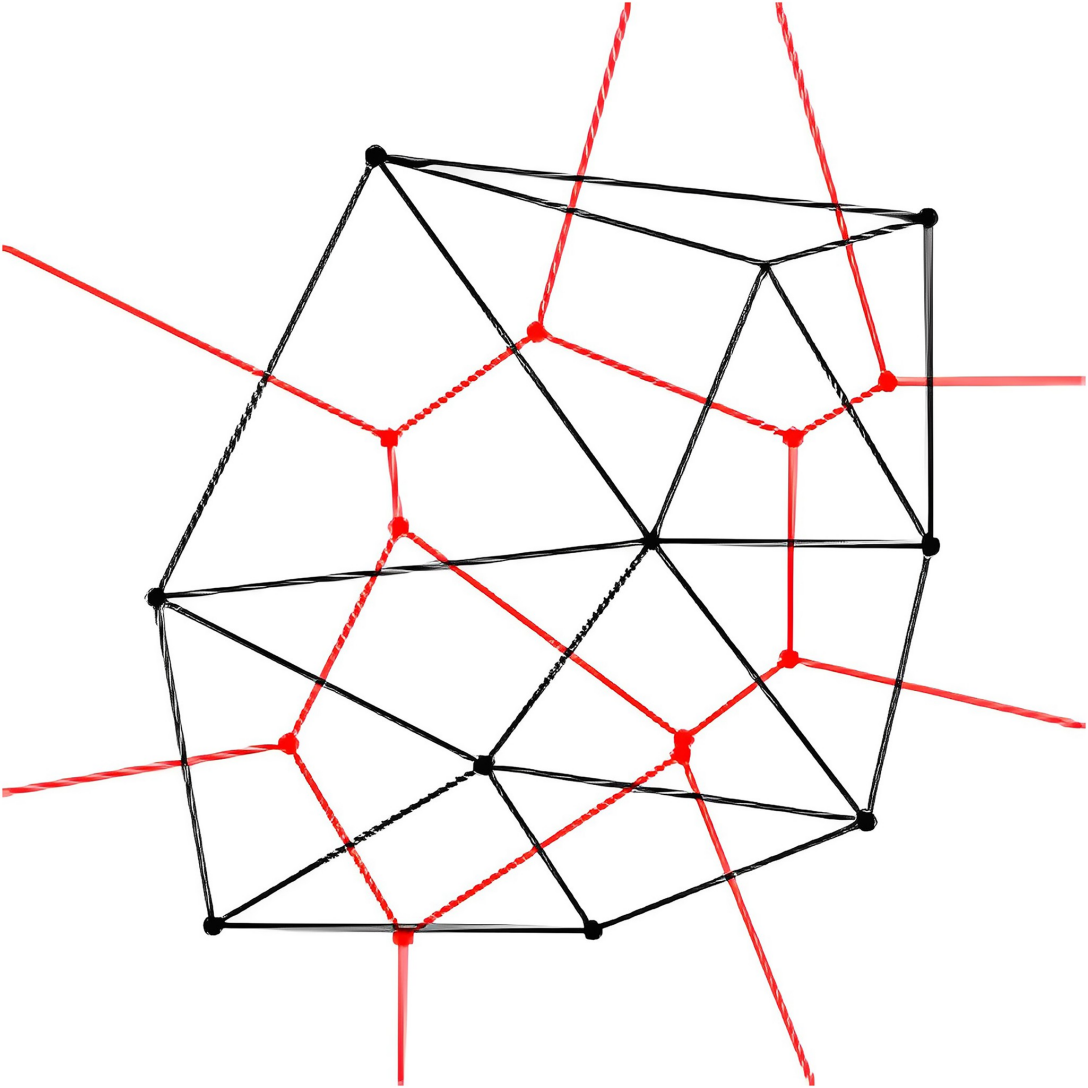
Delaunay triangulation and Voronoi diagram [108].

In the following sense, Delaunay triangulation *D*(*P*) of *P* is the Voronoi diagram’s dual: it contains the same number of points as the Voronoi diagram. A simplex with vertices, *p*_1_ … *p*_*k*_ and an array of *V*_1_ … *V*_*k*_ of Voronoi cells corresponding to point, *p*_1_ … *p*_*k*_ has a nonempty intersection, n, belonging to Delaunay triangulation. It is a simplicial complex derived from the convex hull of the points in P. That is, if the common intersection of the corresponding Voronoi cells is not empty, the convex hull of four points in P defines a Delaunay cell (tetrahedron). Similarly, if the intersection of their corresponding Voronoi cells is not empty and has three or two points, the convex hull denotes as Delaunay face or edge. The Delaunay triangulation and Voronoi diagram are shown in Fig 9.

Voronoi diagram V(P) of P is a convex polyhedron cell decomposition of $R_{3}$. Each Voronoi cell comprises exactly one sample point, and all points of R3 that are not closer to any other sample point, that is, the Voronoi cell corresponding to *p* ∈ *P* is as follows.


$$V_{p}=\{x\;\in R_{3\;}:\forall q\;\in P\;∥x-p∥\leq ∥x-q∥\}$$

Voronoi facets are closed facets shared by two Voronoi cells, Voronoi edges are closed edges shared by three Voronoi cells, and Voronoi vertices are closed points shared by four Voronoi cells. Besides, the mesh can also be computed for TSDF-based approaches based on Marching Cubes [91].

#### Point-based method: Point cloud representation

A cloud point representation refers to a group of recorded depth maps. Point clouds represent the output of numerous 3D sensors like laser scanners and a technique for representing 3D scenes. When a point-based input is turned into a continuous implicit function, it is discretized and then transformed into an (explicit) form through costly polygonization [80] or ray casting [81], then the computational overheads required for switching between several data representations are unveiled. Additionally, using a regular voxel grid, which tightly depicts empty space and surfaces and so severely limits the size of the reconstruction volume, imposes memory overheads.

Moving volume systems [51, 57], which function in extremely low volumes, but which release voxels when the sensor moves, or volumetric hierarchical data structures [82], which incur further computational as well as data structure complexity for a restricted spatial gain, have been developed as a result of these memory limitations. Simpler representations have also been investigated in addition to volumetric techniques.

The input obtained from depth/range sensors are more suited for point-based representations. In the case of real-time 3D reconstruction, [33] used a point-based technique and a custom-structured light sensor. In addition to reducing computational complexity, point-based methods lower the overall memory associated with volumetric approaches (standard grid) as long as overlapping points are combined. Therefore, such strategies were employed in larger reconstructions. However, an obvious compromise in scale versus speed and quality becomes apparent.

**Fig 10:**
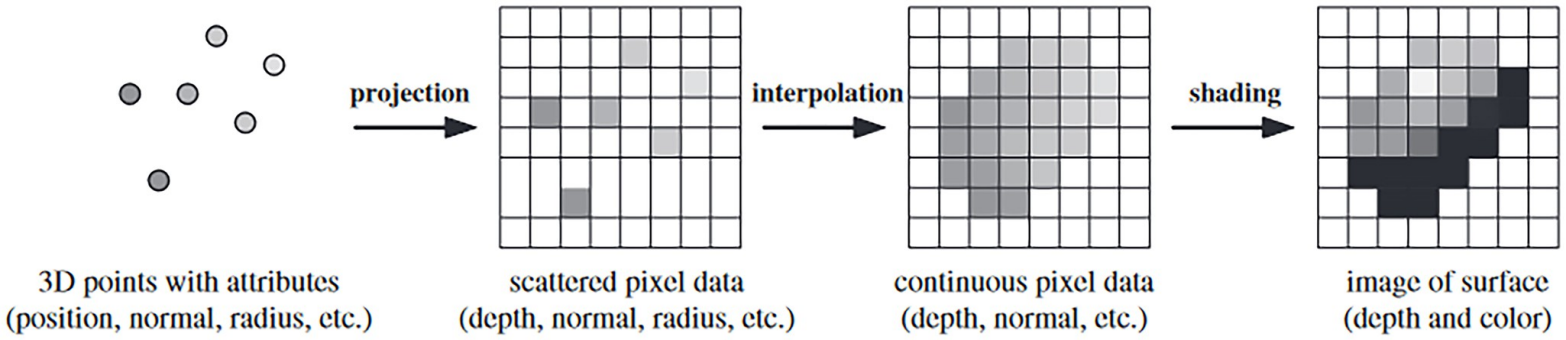
Flow of data on the process of point-based surface rendering [109].

The flow data on the process of point-based surface rendering, as in Fig 10, starts with 3D with attributes such as position, normal, radius, etc. Then, projecting the 3D points into scattered pixel data could give the depth, normal or radius value. The interpolation and shading process would result in the image of the surface with depth and colour information. [85] demonstrated real-time 3D reconstruction using a point-based approach and a customized structured light sensor. Apart from lowering computational complexity, point-based methods have a lower memory footprint than volumetric (regular grid) alternatives, if the points that overlap are combined. As a result, these techniques have been applied to larger-scale reconstructions. Nevertheless, a clear trade-off between scale and speed, and quality becomes apparent. Point-based methods might also be more computationally demanding in terms of storage than compact index-based volume representations based on Marching Cubes. The approach is compact and readily manages data, which can benefit telepresence, which requires instant transmission and fast and compact data structures to reconstruct and provide remote users a virtual 3D model in real-time.

### Evaluation of 3D reconstruction method for telepresence system

Evaluating the 3D reconstruction method is a challenging task. This is not just owing to the increased complexity of the problem but also the absence of widely acknowledged standardized testing procedures. A performance evaluation system in this area is lacking without consideration of the design of experimental test beds and analysis methodologies, as well as the definition of the ground truth. Furthermore, to establish valid objective comparisons, the performance must be quantified and qualified in some way.

#### Performance analysis

For performance assessment, [6, 16, 51, 55, 57, 70, 74, 77, 90] measured frame rates of application and network latency by [6, 17, 91]. [58, 78] monitor the refresh rates of the 3D reconstruction method and the latency of the overall system [15, 42, 51, 70, 75, 76, 78, 92, 93]. [79] measure the processing time for encoding silhouette images. [81] compares average kernel times of IBVH. [41, 84, 86] measure the rendering rate. [4] measure the frame rate for the display. [40, 54, 89] evaluate the speed of modelling. [43, 55, 74, 77, 86, 94] measure the processing time and frame rate of the enhancement method. [16, 42, 51, 52, 70, 75, 83, 88, 93] measure the computational time. The number of mesh faces is calculated by [57, 85] record the sequential alignment comparison. [87] measure the root mean square error (RMSE) of measurements and [70] calculate the number of resultant vertices. The bandwidth for streaming the data to the remote place was done by [17, 91].

#### Visual quality

The visual quality evaluation that has been conducted by [79] measures the temporal qualities of the acquisition result, while [80, 83] compare the quality of the obtained results with a dataset. [4, 41] made comparisons of temporal noise for their results. [19, 37, 42, 57, 94, 110] made a comparative result with respect to the visual quality of the reconstructed model. [53] evaluates the quality of the rendering result, and [16, 88] made a qualitative comparison with other different state-of-the-art methods. Peak signal-to-noise ratio (PSNR) is utilized to quantify the nature of the reconstructed compressed image. The higher value of PSNR indicates a better quality of the recreated image [108].

#### User test

The usability testing has been conducted by [6, 55]. conducted an experiment, and at the end of the session, asked the questionnaire consisted of 10 topics that were covered by groups of two to four separate questions that had to be answered using Likert scales with varying orientations regarding the overall experience, usage experience, comprehensibility of body language and gaze communication, acceptance of the apparatus used and the illusion of physical co-presence. [55] have let the participants experience two separate prototypes and made them rate to compare both systems in terms of which one is more accessible, more preferable, or that could make the participant feel more present and feel the presence of another remote user. User studies [43, 58, 70, 93] and pilot studies [15] have been conducted and evaluated. [91] evaluate the practicality of the framework for telepresence in live-captured scenes while [58, 59] evaluate the user experience of the system.

## Discussion

The publication trend indicates that there is an increasing interest in integrating 3D reconstruction with telepresence systems. However, given the importance of the topic and the relatively small number of reports that summarized this field of study have been found. Hopefully, this systematic literature review can be helpful and valuable for other researchers. Overall, this systematic review of the 48 studies helped answer our three research questions.

### RQ1: What are the input data for the 3D reconstruction method?

The input data which has been used for the 3D reconstruction method are images, and video captured using a regular camera, or depth and colour streams acquired using RGB-D sensors. The input device type has been detailed as illustrated in Fig 10. Over the last decade, a new class of cameras has been revolutionized that enables detailed measurement of the three-dimensional geometry of the scene being scanned, overcoming the limitations of conventional colour cameras. These sensors take a thorough per-pixel scene depth measurement, such as the distance between the scene’s points, and store the information. In most cases, these estimated depth values are given to the viewer as either a profound image of the viewable areas of the scene in a two-and-a-half-dimensional shape. RGB-D is the combination of RGB with a depth sensor of this type. This enables the simultaneous capture of scenery and scene geometry at accepted time frame rates using a stream of colour and depth images. Structured light and active infrared (IR) are two different methods used for depth sensing. Structured light involves projecting known patterns onto a scene and analyzing their deformations to calculate depth information. Meanwhile, active infrared (IR) uses emitted and reflected infrared light to obtain depth information. Time of flight (TOF) and stereo depth sensing are two techniques used in computer vision to determine depth information. TOF measures the time it takes for light to travel and return, while stereo depth sensing compares images from two cameras to calculate depth.

A wide range of RGB-D products such as the Microsoft Kinect Xbox 360, Kinect V2, Azure, Intel RealSense Structure Sensor, and the Asus Xtion Pro have been created over the last ten years. Although earlier sensors were costly and only available to a few subject specialists, the range sensors are now everywhere and are even available on mobile devices of the newest generation. Current sensors are tiny, cheap, and accessible to a large audience daily. The availability of inexpensive sensor technology led to a significant leap in research, particularly with regard to more robust static and dynamic methodologies for reconstruction, from 3D scan applications to precise facial and body tracking systems to be integrated with telepresence systems. Table 4 summarize the details of various type of depth sensors.

**Table 4:** Comparison of consumer-grade RGB-D sensors.

Device Specification	Kinect Xbox 360	Kinect V2	Kinect Azure	RealSense Depth Camera D400 Series	ZED Camera	Structure IO	Xtion Pro Live
**Manufacturer**	Microsoft Corporation	Microsoft Corporation	Microsoft Corporation	Intel	Stereolabs	Occipital	ASUS
**Colour Camera**	640×480 pixels @ 30 Hz	1920×1080 pixels @ 30 Hz	3482×2160 pixels @ 30 Hz	1920x1080 pixel @ 30 Hz	1920×1080 pixels @ 30 Hz	640 x 480 pixels @ 100 Hz	640x480 pixels @ 30 Hz
**Depth Camera**	640×480 pixels @ 30 Hz	512×424 pixels @ 30 Hz	1024 x024 pixels @ 15 Hz	1280x720 pixel @ 90 Hz	1280 x 768 pixel @ 100 Hz	1280 x 960 pixel @ 54 Hz	1280 x 1024 pixel @ 60 Hz
**Sensor**	Structured light	Time of Flight (TOF)	Time of Flight (TOF)	Active infrared (IR) stereo	Stereo Depth Sensing	Active infrared (IR)	Active infrared (IR)
**Dimensions: Length x Width x Height**	6 x 6 x 12 inches	10 x 3 x 3 inches	4 x 1.5 x 5 inches	3.5 x 1x 1 inches	6.7 x 1.2 x 1.3 inches	4.3 x 0.7 x 0.9 inches	18 x 3.5 x 5 inches
**Depth Field of View (FOV) (H x V)**	57°x 43°	70° x 60°	75° x 65°	65° x 40°	90° x 60°	59° x 46°	58°x 45°
**OS Required**	Windows 7,	Windows 7,	Windows 7,	Windows 7,	Windows 7,	Windows 7,	Windows 7,
	Windows 8	Windows 8,	Windows 8,	Windows 8,	Windows 8,	Windows 8,	Windows 8,
		Windows 10	Windows 10	Windows 10	Windows 10	Windows 10	Windows 10
**Price**	$ 150.00 (discontinued)	$199.00 (discontinued)	$399.00	$ 179.99	$349.00	$449.00	$300.00

### RQ2: What are the real-time 3D reconstruction methods implemented in telepresence systems?

The 3D reconstruction method for telepresence can be classified into four methods. First is the visibility method most suitable for the traditional computer vision approach using the image or video frame as input and applying the shape-from-silhouette algorithm. The second method is the volumetric method which executes a Truncated Signed Distance Function (TSDF) to generate the surface representation of captured object or environment as a voxel grid in which every voxel records the distance to the nearest area. The third method is the triangulation method which is used for mesh generation. The Delaunay triangulation algorithm is the most used algorithm to generate the mesh of the reconstructed model. Last but not least is the point-based method. This method is mainly preferred as it helps to reduce computational complexity and lower the overall memory associated with volumetric approaches.

Therefore, the telepresence system represents the target objects as sets of 3D volume pixels, or voxels, in a 3D box. The actual environment is then produced dynamically from any viewing angle at the local endpoint, inserting the point cloud object into a scene or rendering many concurrent point cloud objects. Consequently, it requires complicated preprocessing and rendering, including setups with many camera angles and RGB and depth cameras. Moreover, volumetric media is extremely dense because each voxel is transmitted only once. Therefore, a higher level of compression exchanges computation and latency for the bandwidth and latency required for networking, and vice versa. The accuracy of the reconstructed model can affect the telepresence system, as agreed by [56, 58], where the quality of reconstruction with visual cue offset has been directly influencing the user experience while performing remote communication using telepresence.

There is an apparent trade-off between scale, speed, and quality. By comparing the previous and the latest study that has been analyzed in this report, it is apparent that there are continuous improvements in each of the methods as there is also gradual advancement of devices and machines made available for researchers. It is vital to adopt the appropriate reconstruction method to ensure that the accuracy and computation capacity of the reconstructed model can be advantageous when integrated with a telepresence system, resulting in positive user interaction.

### RQ3: How can the real-time 3D reconstruction method be evaluated for the telepresence system or application?

There are several ways to evaluate the overall system of the 3D reconstruction method integrated with telepresence technology. The performance of 3D reconstruction and telepresence components can be quantified using performance analysis, visual quality comparison, and data gathered from user testing. The evaluation of the 3D telepresence system mostly depends on the research’s main objective. Suppose the researchers’ work mainly focuses on improving the quality of the reconstructed model or improving the 3D reconstruction method. In that case, the visual quality comparison and the performance of the 3D reconstruction method implemented are measured as evaluation. When the research works more towards improving the user experience from using the system, then user testing is the proper evaluation.

## Conclusion

This work conducted a comprehensive systematic literature survey to detect and examine the various 3D reconstruction methods scientists use for telepresence. We also present their advantages and disadvantages in the report. 48 literature publications were selected and analyzed through several phases in the systemic review process.

The literature under evaluation has certain restrictions, as articles published between 2010 and 2022 are the literature documents considered in the review. It, therefore, restricts study and gives future research more scope regarding comprehending the devices before 2010. It also limits research. From this systemic review literature, researchers may gain an in-depth understanding and use this material to advance their study in this field for application in real-time 3D reconstruction

## Supporting information

S1 Checklist PRISMA 2009 checklist. (DOC)

S1 Fig PRISMA 2009 flow diagram. (DOC)
