# Supplementary material for: A systematic literature review: Real-time 3D reconstruction method for telepresence system
Source: PLoS One. 2023 Nov 15;18(11):e0287155. doi: 10.1371/journal.pone.0287155 (PMC10651044; doi:10.1371/journal.pone.0287155)
Supplement: S1 Fig — (DOC) [file pone.0287155.s002.doc]

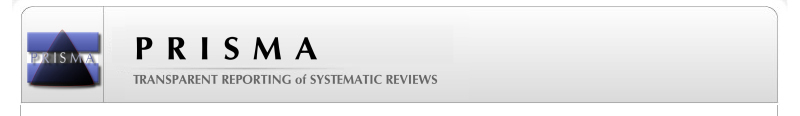
**PRISMA 2009 Flow Diagram**

**Identification**

Records obtained with database searching

*(n =662)*

Additional records identified through other sources

*(n =12)*

**Screening**

**Included**

**Eligibility**

Records after duplicates removed

(n = 314)

Records screened

(n =98)

Records excluded for titles and

abstracts screening

(n =63)

Full-text articles assessed for eligibility

(n = 48)

Studies included in qualitative

synthesis

(n =48)

Records excluded (-348)

(excluded due to double)

Records excluded (-216)

(excluded were mainly systematic review

articles, Non-English literature, books

related, conference proceeding, in

<2010)

Records excluded (n =-35)

(excluded due to screen the titles and abstracts)

Full-text articles excluded, with reasons (n =-15) (excluded due to did not focus telepresence.)
